# Case report: A 17-year-old male with primary pulmonary osteosarcoma

**DOI:** 10.3389/fmed.2024.1364937

**Published:** 2024-03-21

**Authors:** Xin Wen, Liyan Xue, Xu Jiang, Jiuming Jiang, Meng Li, Li Zhang

**Affiliations:** ^1^Department of Diagnostic Radiology, Center for National Cancer, Cancer Hospital, Chinese Academy of Medical Sciences and Peking Union Medical College, Beijing, China; ^2^Department of Pathology, Center for National Cancer, Cancer Hospital, Chinese Academy of Medical Sciences and Peking Union Medical College, Beijing, China

**Keywords:** primary pulmonary osteosarcoma, computed tomography, positron emission tomography, imaging, diagnosis

## Abstract

Primary pulmonary osteosarcoma is one of the extraskeletal osteosarcomas originating from the lung with an extremely low incidence and highly invasive potential. Here we report a case of primary pulmonary osteosarcoma treated in our hospital with a literature review. The patient, a 17-year-old male, had a cough and hemoptysis for 20 days. Computed tomography (CT) and positron emission tomography (PET)/CT were performed in our hospital. According to pathological examination after surgery, the tumor was diagnosed as a high-grade sarcoma with remarkable osteogenesis and necrosis. Based on radiological and histological examinations, a diagnosis of primary pulmonary osteosarcoma originating was considered. The patient underwent surgery and adjuvant chemotherapy. This patient has been under consecutive follow-up for nearly 8 years, showing no signs of recurrence or distant metastasis. Primary pulmonary osteosarcoma is a rare lung malignancy that shows rapid progression, nonspecific symptoms and inapparent signs at an early stage. The diagnosis of primary pulmonary osteosarcoma highly relies on imaging and histological examinations, among which chest CT is the predominant method to check this disease.

## 1 Introduction

Primary pulmonary sarcoma is extremely uncommon with an incidence of one sarcoma for every 500 carcinomas, and primary pulmonary osteosarcoma, a highly malignant soft tissue tumor, is the rarest histological type of primary pulmonary sarcoma (1, 2), therefore it is often misdiagnosed. Consequently, more knowledge is beneficial to the early detection and treatment of this disease, which is also vital to the improvement of prognosis (3, 4). A case of primary pulmonary sarcoma treated in our hospital is reported in this paper with a literature review.

## 2 Case description

A 17-year-old male patient had a cough and hemoptysis for 20 days, a mass in the lower lobe of the left lung was observed during the examination of an external hospital, therefore he came to visit our hospital. His medical history and family history were negative. Laboratory examination demonstrated no abnormalities in the examination of lung cancer tumor markers CA125, cyfra21-1, NSE, SCC, or CEA. In CT images, the lesion appeared as a soft tissue density mass at the lateral bronchus of the dorsal segment of the left inferior lobe, which also grew towards the lumen of the left interlobular pulmonary artery. It was a spherical-like mass with a maximum diameter of 4.2 cm and uneven density. In addition, there were small striations and areas of bone density to the right of the center, and its postero-lateral side appeared hypodense. The mass was ill-defined, with no abnormal density in the surrounding lung tissue, no clear enlarged lymph nodes in the mediastinum, and no abnormal density changes at the rib scan level. The enhancement scan revealed mild to moderate heterogeneous enhancement with poorly defined boundaries and no enhancement in the cystic region. ^18^F FDG PET/CT images demonstrated a tumor in the left lower lobe of the lung with an unevenly increased metabolic rate and increased mediastinal lymph node metabolism with no other systemic PET/CT abnormalities observed, which indicates the high potential of lymph node metastasis and no distant metastasis. This lesion exhibits the obvious absence of elevated metabolism in its postero-lateral side, where the density was relatively low in contrast-enhanced CT. Combining CT and PET/CT assessments, the initial preoperative TNM staging was determined to be T2N1M0.

**Figure 1:**
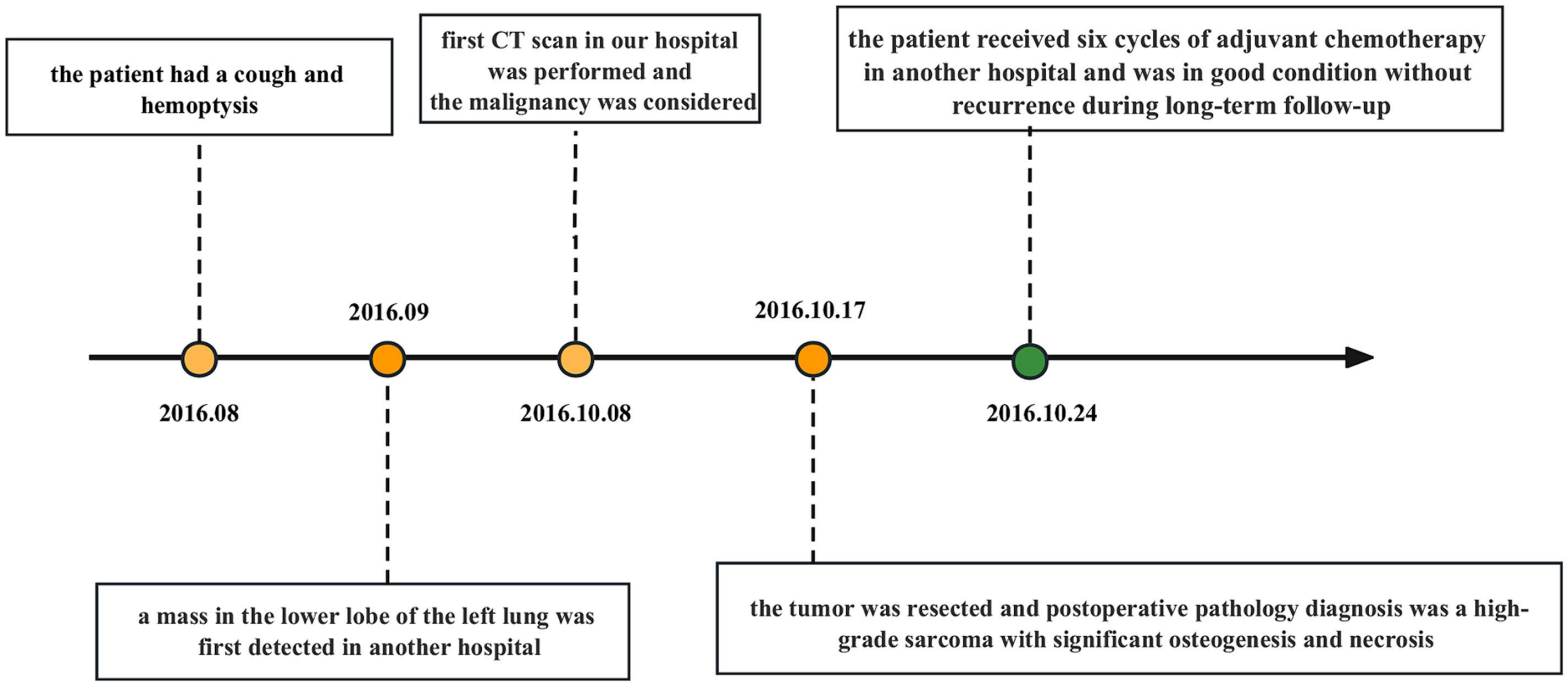
Timeline with relevant data about the onset, diagnosis, and therapy of the patient with primary pulmonary osteosarcoma.

After a comprehensive evaluation, the surgical resection was performed. The dimension of the postoperative lobectomy specimen was 14 × 10 × 3.8 cm. As the lung was dissected along the bronchus, a mass measuring 4.2 × 4 × 3.8 cm with a firm, off-white surface and locally discernible ossification was discovered at the root of the lung. The mass involved the lobar and segmental bronchi and did not involve the visceral pleura. At a maximum diameter of 0.4–1.0 cm, peripheral lungs were grey-red and mushy, with localized lamellar thickening apparent under the surrounding pleura. Pathological diagnosis: the mass in the lower lobe of the left lung was a high-grade sarcoma with significant osteogenesis and necrosis, indicating that it was an extraosseous osteosarcoma based on the morphology and immunophenotype. The tumor had a maximal diameter of 4.2 cm and involved the segmental and lobar bronchi, but not the visceral pleura and lymph nodes. The immunohistochemistry results showed AE1/AE3 (−), EMA (−), Vimentin (3+), Bcl2 (1+), CD99 (2+), Ki67 (40%+), SMA (−), Desmin (−), TTF1 (a few scattered cells+), CD34 (−), S100 (−), Calponin (−). The patient underwent surgery for osteosarcoma resection, along with lymph node clearance. Subsequently, he received six cycles of systemic chemotherapy with doxorubicin at a dose of 25 mg/m^2^ (day 1–day 3) and cisplatin at a dose of 100 mg/m^2^ (day 4), with one cycle consist of 21 days. After treatment, the patient’s vital physical signs and health conditions remained stable. This patient has been under consecutive follow-up for nearly 8 years, showing no signs of recurrence or distant metastasis which are assessed by CT or MRI. The timeline of patient’s diagnosis, treatment and follow-up has been displayed in Figure 1.

## 3 Discussion

Primary pulmonary osteosarcoma is a relatively rare tumor that originates in the lung and accounts for only 0.01% of malignant tumors (3). There have only been 31 cases of primary pulmonary osteosarcoma reported (Table 1). So far, no confirmed etiology for the disease has been found, and radiation therapy administered near the lesion’s location and trauma may contribute to primary intrapulmonary osteosarcoma’s development (1). Takamura et al. (23) reported a case of extraosseous osteosarcoma secondary to chemoradiotherapy in the lung. But in this case, there was no history of such treatment in this patient.

**Table 1:** Reported cases of primary pulmonary osteosarcoma.

Author	Age	Gender	Chief complaints	Size and location	Procedure to make definite diagnosis	Calcification on CT scan	Bone scintigraphy uptake	Treatment	Prognosis
Greenspan (5)	35	F	Pain, hemoptysis	Left main bronchus, 7 cm	Autopsy	ND	ND	No radical treatment	DOD 11 M after symptoms onset
Yamashita et al. (6)	74	F	Progressive asthmatic symptoms	Large mass replacing left lung	Autopsy	ND	ND	No radical treatment	DOD 6 M after symptoms onset
Reingold and Amromin (7)	62	M	Pneumonia	Right middle lobe, 6 × 6 × 4 cm	Necropsy	ND	ND	Chemotherapy	DOD 7 M after symptoms onset
	56	F	Chills, fever, chest pain	Left upper lobe, 7 × 5 × 4 cm	Surgery	ND	ND	Surgery	Alive 14 M after surgery
Nosanchuk and Weatherbee (8)	66	M	Weakness dyspnea, chest pain, and hemoptysis	Entire left upper lobe and most of lower lobe	Autopsy	ND	ND	No radical treatment	DOD 4 M after symptoms onset
Nascimento (9)	77	F	Asymptomatic	Right middle lobe, 4 cm	Surgery	ND	ND	Surgery	DUC 6 M after surgery
	72	M	Asymptomatic	Right middle lobe, 5.5 cm	Surgery	ND	ND	Surgery	DUC 10 M after surgery
Bagaric and Belicza (10)	49	F	ND	Right lower lobe	ND	ND	ND	ND	DOD
Saito et al. (11)	83	M	Chest pain	Right middle lobe 10 cm	ND	ND	ND	ND	DOD 4 M after symptoms onset
Colby et al. (12)	61	M	Respiratory symptoms	Right lung, 16 × 10	ND	ND	ND	ND	Died several months later of unknown causes
	51	M	Cough	Left lower lobe, 11 × 7 cm	ND	ND	ND	ND	Alive 6 M after surgery
	77	F	Pneumonia	Right middle lobe, 4 × 3 cm	ND	ND	ND	ND	DUC 6 M after surgery
Loose et al. (13)	54	M	Chest pain, left upper extremity paresthesia	Left upper lobe, 10 cm	Surgery	(+)	Performed after surgery	Surgery, chemotherapy, radiation	Alive 7 M after surgery
	45	F	Chest pain	Left lower lobe, 5.5 cm	Surgery	(−)	ND	Surgery	Alive 2 M after surgery
Petersen (14)	70	M	Asymptomatic	Left lower lobe, 5 × 3.5 × 6 cm	Surgery	(+)	(+)	Surgery, radiation	Alive 6 M after surgery
Stark et al. (15)	59	M	Asymptomatic	Left lower lobe, 6.5 × 7 × 11 cm	Surgery	(+)	ND	Surgery	ND
Bhalla et al. (16)	58	M	Fever and cough	Left upper lobe, 18 × 9 × 8 cm	Autopsy	(+)	ND	No radical treatment	DOD about 1 M after first admission
Miller and Allen (17)	72	M	ND	ND	TBLB, splenectomy	ND	ND	Chemotherapy, radiation	DOD 12 M after symptoms onset
Sievert et al. (18)	56	M	Tingling in left fingertips	Left upper lobe, 4 × 2 × 2 cm	Surgery	(−)	Performed after surgery	Surgery	Alive 12 M after surgery
Chapman et al. (4)	33	F	Cough, left-sided chest pain	Left lung, 5.5 × 5 × 4 cm	Surgery	ND	(+)	Surgery, chemotherapy	Alive 42 Mafter surgery
Magishi et al. (19)	74	F	Asymptomatic	Left upper lobe, 5.7 × 5 × 3.3 cm	Surgery	(−)	Performed after surgery	Surgery	DOD 11 Mafter surgery
Kadowaki et al. (2)	72	M	Chest pain, dyspnea	Left lower lobe, 9 × 9 cm	Needle biopsy	(+)	(+)	ND	DOD 3 M after symptoms onset
	77	M	Hemoptysis	Left lower lobe, 11 × 8 cm	Needle biopsy	(+)	(+)	ND	DOD 3 M after symptoms onset
Yamazaki et al. (20)	73	M	Cough, hemoptysis	Left upper lobe, 7 × 6.5 cm	ND	ND	ND	ND	DOD 7 M after operation
	77	M	Hemoptysis	Left lower lobe, 11 × 8 cm	ND	ND	ND	ND	DOD 3 M after symptoms onset
Gu et al. (21)	58	M	Chest pain	Right lung, 5.7 × 8.0 × 5.3 cm	Surgery	(+)	(+)	Surgery, chemotherapy	ND
Shenjere et al. (22)	66	M	Found on assessment for trapped nerve	Left upper lobe, 6 × 5 cm	Biopsy	ND	ND	Surgery	Alive 12 M after initial symptoms
	79	M	Shortness of breath	Left upper lobe, 11 × 9 cm	Biopsy	(−)	(+)	Radiation	DOD 5 M after initial symptoms
	56	F	Cough	Right lower lobe, 3.2 × 2.2 × 2 cm	ND	ND	ND	ND	ND
	58	F	Chest pain, cough	Left lower lobe, 25 × 24 × 6 cm	ND	ND	ND	ND	ND
Takamura et al. (23)	80	M	ND	Right middle lower lobe, 14 cm	Autopsy	ND	ND	Radiotherapy, chemotherapy	DOD
Current case	17	M	Cough and hemoptysis	Left lower lobe, 4.2 × 4 × 3.8 cm	Surgery	(+)	(+)	ND	ND

DOD, died of disease; DUC, died of unrelated cause; M, month; ND, not documented; TBLB, transbronchial lung biopsy.

In contrast to osteosarcoma originating from the bone which tends to affect younger patients, primary pulmonary osteosarcoma mostly affects patients over the age of 50, with a male-to-female ratio of 1.9:1 according to previous case reports. But in this case, we reported a 17-year-old case of primary pulmonary osteosarcoma, and as we know, this is the youngest patient reported to date.

The clinical manifestations have always been unremarkable, with the primary symptoms being chest pain, cough, and hemoptysis (3, 24–26). Among the published reported cases (including the current one), the left lung was affected in 21 cases, the right lung in 11 cases, and the upper lobe of the left lung in 12 cases (Table 1).

Imaging and pathological examinations are the key methods used to determine the diagnosis of primary pulmonary osteosarcoma. Extraskeletal osteosarcoma frequently presents on radiographs as soft tissue opacity with various degrees of mineralization. The primary examination method for this disease is a chest CT scan, which typically reveals a large, lobulated soft tissue mass in the lung with irregular stripes and nodular dense calcifications inside and around the lung, as well as intact neighboring bone structures and no signs of destruction. When necrosis and hemorrhage occur within the tumor, the enhanced scan is typically unevenly enhanced. Calcification or osteoid matrix formation occurs in approximately 50% of primary lesions and can appear during the course of the disease or worsen over time, which also appeared in the images of this case. Calcification on CT scan was clearly indicated in 26% (8/31) of the reported cases (Table 1). PET/CT showed increased metabolic rate of this mass (21). Under the microscope, malignant, primitive spindle cells and osteoid matrix are the hallmarks of primary pulmonary osteosarcoma’s pathology (26). A lung mass must meet the following diagnostic criteria to be considered primary lung osteosarcoma: (1) the tumor must consist of a uniform pattern of osteosarcomatous tissue; (2) the tumor must produce osteoid or bone matrix; and (3) the tumor must be originated from the lung and exclude the possibility of a primary osteogenic tumor (26).

**Figure 2:**
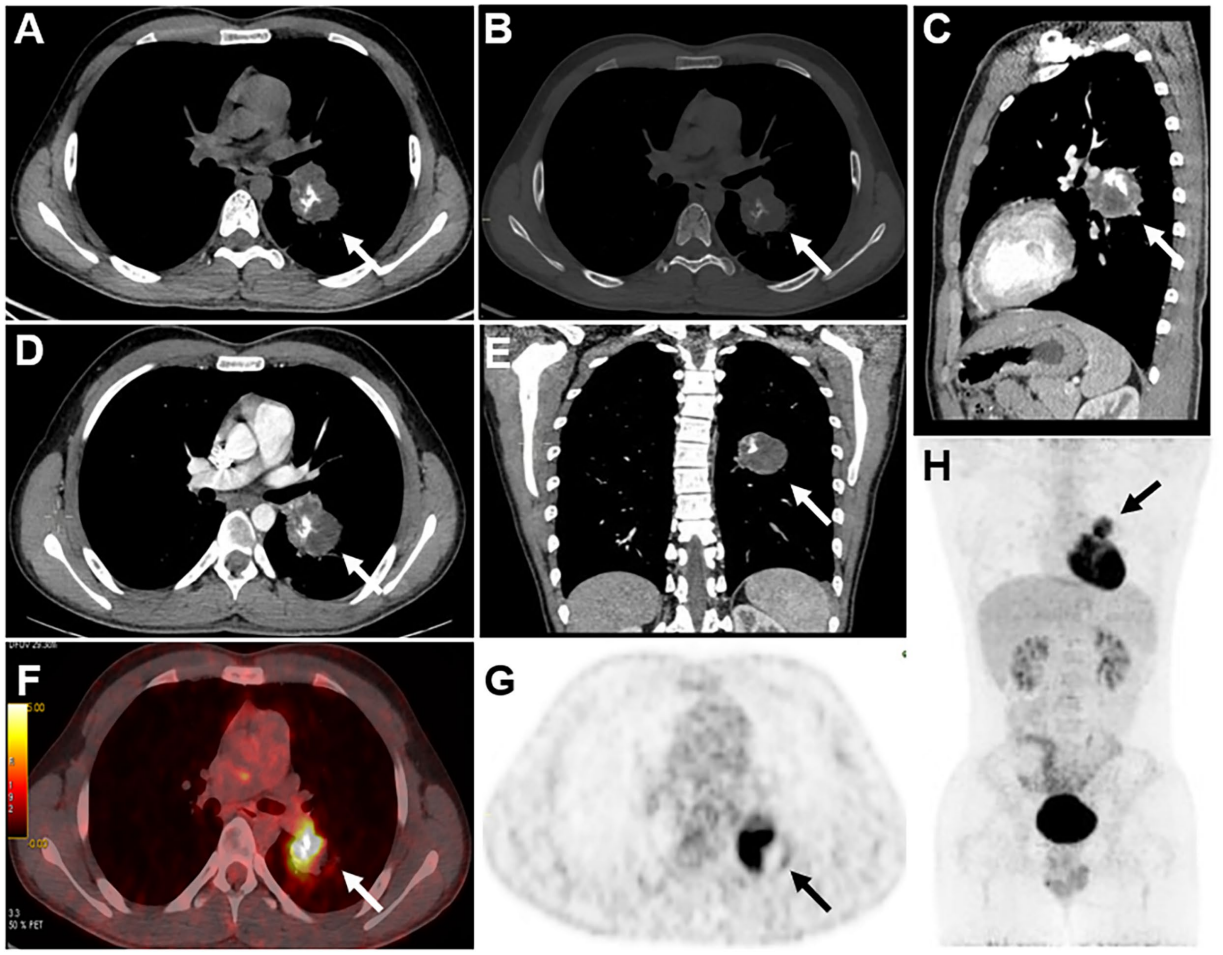
CT scans of the mediastinal window **(A)**, bone window **(B)**, and sagittal mediastinal window **(C)** revealed a mass-like soft tissue density shadow in the dorsal segment of the lower lobe of the left lung. The mass had an irregular sphere-like margin, uneven density within the mass, and irregular eccentric ossification or calcification. In addition, axial **(D)** and coronal **(E)** enhanced CT images also revealed a heterogeneous mass with no enhancement in the cystic portion. ^18^F-FDG PET/CT revealed uneven increased metabolism of the mass in the lower lobe of the left lung, with barely absent uptake in its postero-lateral side **(F,G)**, and the area in the postero-lateral side with increased metabolic activity in lymph nodes was also observed, which also appears hypodense on contrast-enhanced CT. No elevated metabolic activity was observed in other areas on the PET/CT scan **(H)**. Based on a comprehensive evaluation of CT and PET/CT findings, the initial staging before surgery was T2N1M0.

**Figure 3:**
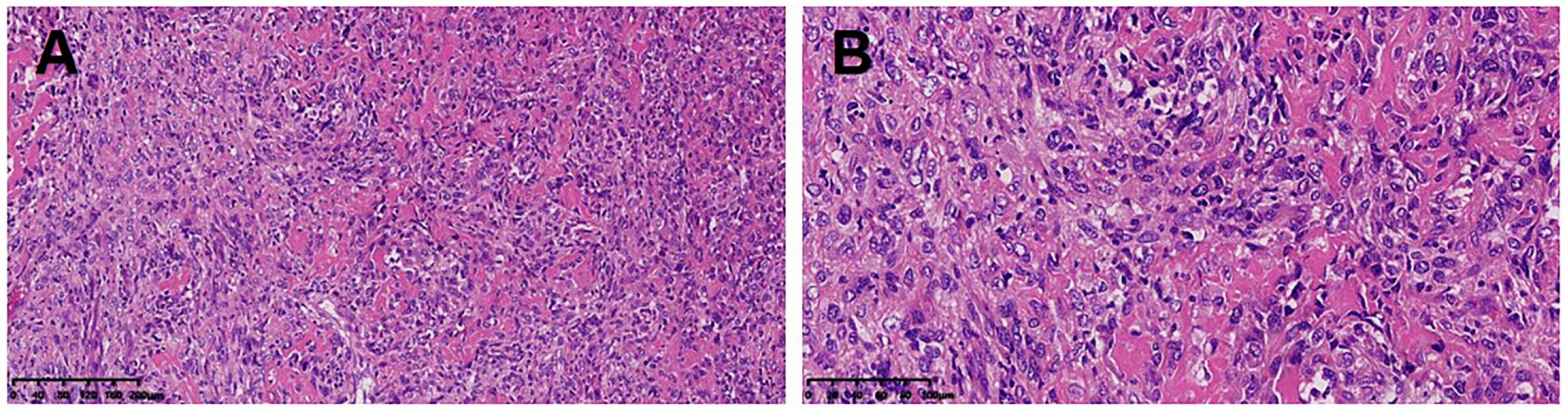
Malignant, primitive spindle cells and osteoid matrix were observed **(A,B)**.

Several differential diagnoses should be considered when a primary pulmonary osteosarcoma is suspected. Clinically, primary pulmonary osteosarcoma first needs to be separated from pulmonary metastases of osteosarcoma that originated from bone. The lung is the principal location of metastasis of osteosarcoma, which typically occurs in adolescents, and primary pulmonary osteosarcoma mainly predominates in middle-aged people and elders. One of the key methods for distinguishing between the two is a whole-body bone scintigraphy, which can identify high uptake changes in the primary bone lesion while also ruling out primary osteosarcoma elsewhere in the body, particularly in the chest wall and nearby ribs. Additional disorders that require differentiation include primary lung cancer with substantial intrapulmonary calcifications, intrapulmonary hamartoma and benign pulmonary calcifications (2, 27). The key points of differentiation in terms of imaging are as follows. Multiple round variable-sized nodules and diffuse interstitial thickening are typical radiologic findings of pulmonary metastasis (28). Stratified or annular calcification is usually harmless which occurs more frequently in granulomas or tuberculous lesions. Hamartomas are characterized by popcorn calcifications (29). None of the above features were present in this patient (see Figures 2, 3).

The preferred method of treatment, which has been applied in 12 of the cases that have been recently described, is surgical removal of the primary tumor, and there are relevant studies suggesting that compared with En bloc resection or extensive surgery, inadequate resection was referred to as poor survival (14). While the effectiveness of chemotherapy and radiotherapy is still debatable. In terms of prognosis, it is difficult to predict the prognosis of primary pulmonary osteosarcoma because of its rarity, but the prognosis appears to be poor. Of the reported cases, 15 patients died of their own disease within 1 year and 4 died of unrelated causes, with the longest survival being 42 months after total pneumonectomy, but this patient developed extensive metastases. All the above cases indicated that this disease typically has a relatively poor prognosis, however, in this case, there has been no evidence of metastasis or recurrence during an 8 years follow-up period. This may perhaps be related to his relatively younger age and timely treatment. The prognosis may also be impacted by the greatest tumor diameter, the presence of calcification, insufficient surgical resection, and local recurrence. Nascimento et al. considered that the prognosis is bad when the maximum tumor diameter is more than 5 cm. Benign indicators are thought to be the presence of osteoid in microscopic results and calcification in imaging (2, 3, 14).

Additionally, we should point out that although the final diagnosis was primary pulmonary osteosarcoma, we highly suspected that it was originating from the left interlobular pulmonary artery for the reason that it was located at the lateral bronchus within the dorsal segment of the left inferior lobe and extended towards the lumen of the left interlobular pulmonary artery, and Zhai et al. (26) once reported a case of osteosarcoma originating from the pulmonary artery. But in this case, the possibility that the tumor originated in the lung and later invaded the pulmonary arteries cannot be excluded completely, which is difficult to differentiate by the imaging examination.

In summary, primary pulmonary osteosarcoma is an extremely scarce malignancy that is associated with a high incident rate of lymphatic and hematogenous metastasis, and early diagnosis and treatment are crucial to the patient’s prognosis. We can identify original intrapulmonary osteosarcoma using imaging and histopathological findings. To be more specific, imaging helps to diagnose the lesion and make a differential diagnosis by giving a clear visual of the lesion. A CT scan of the lung typically displays a large, lobulated soft tissue mass with irregular stripes and nodular dense calcifications inside and around the lung, as well as intact neighboring bone structures and no signs of destruction. The enhanced scan is often unevenly increased when necrosis and bleeding occur within the tumor. PET/CT is commonly performed in oncology patients to exclude distant and lymph node metastasis, perform neoplasms staging and evaluate treatment response. Tumor cells typically exhibit heightened metabolic activity, and increased uptake of radiopharmaceuticals in PET/CT suggests the potential for metastasis (30). The histopathological identification of this tumor is based on the microscopically observed tumor cells and osteoid matrix (4, 26).

## 4 Patients’ perspective

Our patient states that upon identifying the mass in the lower lobe of my left lung, the medical team promptly conducted a surgical procedure, providing me with a corresponding postoperative plan. As of now, my condition has been favorable.

## Data availability statement

The original contributions presented in the study are included in the article/supplementary material, further inquiries can be directed to the corresponding authors.

## Ethics statement

The studies involving humans were approved by Ethics Committee of Cancer Hospital, Chinese Academy of Medical Sciences. The studies were conducted in accordance with the local legislation and institutional requirements. The participants provided their written informed consent to participate in this study. Written informed consent was obtained from the individual(s) for the publication of any potentially identifiable images or data included in this article.

## Author contributions

XW: Formal analysis, Writing – original draft. LX: Formal analysis, Writing – review & editing. XJ: Writing – original draft. JJ: Formal analysis, Writing – review & editing. ML: Formal analysis, Funding acquisition, Supervision, Writing – review & editing. LZ: Formal analysis, Funding acquisition, Supervision, Writing – review & editing.
